# Correction: Severe COVID-19 infection: An institutional review and literature overview

**DOI:** 10.1371/journal.pone.0310284

**Published:** 2024-09-06

**Authors:** The PLOS ONE Editors

## Notice of Republication

This article was republished on August 23, 2024, to address an issue identified post-publication. An updated version of S1 File is provided with this notice. Please download this article again to view the correct version.

The article’s Data Availability statement is updated to: All relevant data are within the paper and its Supporting Information files.

## Supporting information

S1 File Raw data file. (XLSX)
