# Supplementary material for: Severe COVID-19 infection: An institutional review and literature overview
Source: PLoS One. 2024 Aug 20;19(8):e0304960. doi: 10.1371/journal.pone.0304960 (PMC11335168; doi:10.1371/journal.pone.0304960)
Supplement: Attachment — Submitted filename: Response to reviewers plos one 12.23.23.docx [file pone.0304960.s002.docx]

December 23, 2023,
Editor in Chief

PLOS ONE

Subject: Submission of revised manuscript (PONE-D-23-21699)

Dear Dr. Chen,

We are pleased to re-submit a revised version of our manuscript titled, “**Severe COVID-19 Infection: An Institutional Review and Literature Overview**”.

Thank you for the opportunity to revise and resubmit this manuscript. According to the deadline provided, I am submitting this revision before 01/15/2024. We would like to thank you and the reviewers for the time taken to review our manuscript, and the insightful recommendations that were provided. We have incorporated the suggested changes into the manuscript as much as possible. We look forward to working with you and the reviewers to move this manuscript closer to publication in PLOS ONE.

We have tracked any changes made to the manuscript document. We have specifically responded to each reviewer’s comments below. We have numbered each reviewer’s comment, followed with a response detailing how we have addressed the comment.

Review Comments to the Author

**Reviewer #1:** Congratulations on the work and the paper. It seems everything has been addressed to the slightest detail

Response to review #1

Thank you for your thoughtful review. We appreciate your time and effort in evaluating our work. We are delighted to hear that you found our paper comprehensive and that we have successfully addressed the details to your satisfaction.

**Reviewer #3**: Congratulations to address COVID-19 risk factors and the importance of being vaccinated.

Your study is well written but there is too much information. Despite being a observational study ,the authors conduct a detailed review of all the variables shown in the results section, giving the impression for the reader that we are reading a narrative review. It is important to adequate the lenght of the writing to not become tiring to the reader. The discussion section should point out the main topics to guide the reader through the authors point of view to support their findings. I suggest to reduce the discussion section as the core of the paper is a observational study and their findings. I suggest put table 6 ( medication ) in supplemental matherial and point out in the results and discussion section the most relevant points in this section. I suggest put the limitations and trengths section before the conclusion .

Response to reviewer #3
Thank you for your thoughtful comments. We appreciate your recognition of the detailed overview that all the authors have carefully worked together to collate.

Our aim is to present an unbiased portrayal of our findings, including both supportive and opposing studies. We have also provided possible explanations for discrepancies and believe our comprehensive approach offers valuable insights in a neutral manner. We value your input and the time dedicated to enhancing our paper. We have significantly condensed the discussion by eliminating less relevant findings, removing over two thousand words and 28 references. Additionally, we have placed Table 6 in the supplemental material file, referencing it appropriately in the main text, and reorganized the limitation and conclusion sections as suggested. We hope that these changes improve the manuscript’s readability while maintaining a thorough coverage of this topic.

Sincerely,

**Ogheneyoma Akpoviroro, MD**

Geisinger Northeast/Geisinger Wyoming Valley Medical Center

1000 East Mountain Boulevard

Wilkes Barre, Pennsylvania 18711

United States

570-808-3746

[oakpoviroro@geisinger.edu](mailto:oakpoviroro@geisinger.edu)

[o.akpoviroro@gmail.com](mailto:oakpoviroro@geisinger.edu)
